# Genome-wide identification of F-box proteins in *Macrophomina phaseolina* and comparison with other fungus

**DOI:** 10.1186/s43141-021-00143-0

**Published:** 2021-03-24

**Authors:** Md. Abu Sadat, Md. Wali Ullah, Kazi Khayrul Bashar, Quazi Md. Mosaddeque Hossen, Md. Zablul Tareq, Md. Shahidul Islam

**Affiliations:** Basic and Applied Research on Jute Project, Bangladesh Jute Research Institute, Manik Mia Avenue, Dhaka, 1207 Bangladesh

**Keywords:** F-box proteins, *M. phaseolina*, Disease development

## Abstract

**Background:**

In fungi, like other eukaryotes, protein turnover is an important cellular process for the controlling of various cellular functions. The ubiquitin-proteasome pathway degrades some selected intracellular proteins and F-box proteins are one of the important components controlling protein degradation. F-box proteins are well studied in different model plants however, their functions in the fungi are not clear yet. This study aimed to identify the genes involved in protein degradation for disease development in the *Macrophomina phaseolina* fungus.

**Results:**

In this research, *in silico* studies were done to understand the distribution of F-box proteins in pathogenic fungi including *Macrophomina phaseolina* fungus. Genome-wide analysis indicates that *M. phaseolina* fungus contained thirty-one F-box proteins throughout its chromosomes. In addition, there are 17, 37, 16, and 21 F-box proteins have been identified from *Puccinia graminis, Colletotrichum graminicola, Ustilago maydis*, and *Phytophthora infestans*, respectively. Analyses revealed that selective fungal genomes contain several additional functional domains along with F-box domain. Sequence alignment showed the substitution of amino acid in several F-box proteins; however, gene duplication was not found among these proteins. Phylogenetic analysis revealed that F-box proteins having similar functional domain was highly diverse form each other showing the possibility of various function. Analysis also found that MPH_00568 and MPH_05531 were closely related to rice blast fungus F-box protein MGG_00768 and MGG_13065, respectively, may play an important role for blast disease development.

**Conclusion:**

This genome-wide analysis of F-box proteins will be useful for characterization of candidate F-box proteins to understand the molecular mechanisms leading to disease development of *M. phaseolina* in the host plants.

**Supplementary Information:**

The online version contains supplementary material available at 10.1186/s43141-021-00143-0.

## Background

Protein turnover is the balance between protein synthesis and protein degradation. This process is an important regulator of functioning of the different cellular processes including cell cycle, metabolic control, physical development, and various signal transduction pathways [1, 2]. Protein turnover processes are the same in different cells, however highly different in the aspect of turnover control and regulation [3]. Proteins serve a variety of functions within cells and also interact with other proteins, lipids carbohydrates, and even with DNA [4–7]. Most plants and microorganisms can synthesize proteins inside the cell but animals need to take protein through their daily meal [8]. Protein levels are an important regulator of eukaryotic cell development [9]. Protein synthesis is concluded either by biosynthesis or chemical synthesis procedure.

Proteolysis is the breakdown of protein into smaller polypeptides or amino acids. It is a highly specific process where proteins are hydrolyzed to their specific amino acids [1]. In this process, a diverse group of enzymes and designated proteases is involved. In eukaryotic cells, two major pathways are involved in protein degradation: the lysosomal proteolysis pathway and ubiquitin–proteasome pathway [10]. In the lysosomal proteolysis pathway, cell uptake degraded protein by lysosomes through a non-selective process, but it may become selective during starvation especially under carbon and nitrogen starvation condition [11]. Proteins which are degraded in lysosomal proteolysis pathway are commonly long lived, but their necessities and number are very low. However, most of the intracellular proteins are degraded by another pathway, namely the ubiquitin–proteasome pathway. This pathway is highly specific and targets cytosolic and nuclear proteins for rapid degradation [10, 12].

Ubiquitin is a regulatory protein that is highly conserved in all eukaryotes. Protein degradation under the ubiquitin–proteasome pathway involves three major steps: (i) ATP-dependent activation of ubiquitin by E1 enzyme (ubiquitin-activating enzyme), (ii) transfer of activated ubiquitin to E2 (ubiquitin-conjugating enzyme), and (iii) transfer of ubiquitin to the protein to be degraded by E3 complex (ubiquitin protein ligase) [13]. Polyubiquitinated proteins are recognized by the 26S proteasome in which the targets are specifically degraded. E3 ligases are the F-box protein which form a subunit of SCF complex (Skp, Cullin and F-box containing) and confer specificity for a substrate to be degraded [12]. Studies showed that F-box proteins contain a novel motif, linked to cyclin F along with cell cycle regulators of yeast Cdc4p and Skp2p to Skp1p, which are major components of E3 ligase [14].

The F-box protein was first described as cyclin F in human genome; however, a large number of this protein family exists in different model organisms having various functions [15]. F-box proteins were identified as SCF components; they function as non-SCF complexes, too [16–18]. The number of F-box proteins are greatly varying in eukaryotic organisms and found to be comparatively higher in plants due to diverse functions including physical growth and development, floral organogenesis, senescence, and pathogen resistance [19]. In hemibiotrophic fungus *Magnaporthe oryzae*, F-box protein is crucial for conidiogenesis, fungal growth and development, and finally for virulence [20–22]. In addition, F-box proteins were reported to be involved in sexual reproduction, morphogenesis and for disease-causing ability in human pathogen *Cryptococcus neoformans, Aspergillus nidulans*, and in *Candida albicans* [1, 23, 24]. The understanding of SCF E3 ligases has largely come from extensive studies in two model yeasts, *S. cerevisiae* and *Schizosaccharomyces pombe*. *S. cerevisiae* has at least 20 proteins containing an F-box domain, and several have been well studied, including glucose repression resistant 1 (Grr1) [15]. Despite extensive studies in both model yeasts, very limited studies of SCF E3 ligases have been reported in other fungi. Recent studies on the function of F-box proteins in pathogenic fungi have revealed that SCF E3 ligases are required for fungal virulence. Because of the proven therapeutic potential of the ubiquitin–proteasome pathway for human diseases [25], it would be very important to understand the molecular basis of how this pathway regulates fungal virulence.

Jute is the second most important natural fiber crop after cotton in Bangladesh and called the golden fiber of Bangladesh because of earning a lot of foreign currency [26]. Recently, raw jute and jute product has been considered as the second foreign earning of Bangladesh [27]. However, jute is affected by several biotic and abiotic stresses throughout its growing season and causing yield loss [28]. *Macrophomina phaseolina* is one of the most important pathogens of jute plant causing stem rot disease leading to yield loss up to 30% [29]. This pathogen has more than 500 hosts including food crops, pulse crops, jute, cotton, and also other crops. This fungus can solely reduce up to 30% jute yield among the total production loss due to fungal diseases and others [29]. Consider the economic importance of this fungus genomic information has been carried out to understand its high survivability and disease-causing ability.

Based on the importance of F-box protein in eukaryotes, we have systematically performed the bioinformatic analysis to identify the gene structure, sequence alignment, phylogenetic relationship, exon–intron structures, domain of F-box protein in the stem rot fungus *M. phaseolina*. These results provide an essential understanding of F-box protein in *M. phaseolina* and constitute a strong foundation for further investigation in regulation of fungal virulence, which may lead to novel approaches in developing new antifungal agents.

## Methods

### Identification of F-box proteins from different fungi

To identify the F-box protein sequences in *M. phaseolina*, their protein and genome sequence were downloaded from the website of Basic and Applied Research on Jute Project (BARJ) (http://www.jutegenome.org/gb2/gbrowse/mph/) and NCBI database (https://www.ncbi.nlm.nih.gov/bioproject?term=PRJNA78845). And another online database, comparative fungal genomic platform (CFGP 2.0) (*http://cfgp.riceblast.snu.ac.kr*) was used for the identification of F-box proteins from the selected fungal genomes [30]. In this analysis, a total of 25 fungal genomes (Table 1) were used where Inter Pro domain (IPR001810; IPR006527; IPR007397; IPR012885; IPR013187; IPR017451, and IPR022364) was used as reference for this search. The E-value threshold was selected at 10^-3^ to get the entire possible F-box protein candidates.

**Table 1:** Presence of F-box proteins in different economically important fungi and plants

Species	Kingdom	Lifestyle	No. of F-box protein (total gene)	Source
*Blumeria graminis*	Fungi	Biotroph	24 (6470)	This study
*Puccinia graminis*	Fungi	Biotroph	17 (20,567)	This study
*Melampsora laricis-populina*	Fungi	Biotroph	19 (16,694)	This study
*Magnaporthe oryzae*	Fungi	Hemibiotroph	24 (12,991)	Shi et al. [21]
*Colletotrichum graminicola*	Fungi	Hemibiotroph	37 (12,022)	This study
*Ustilago maydis*	Fungi	Hemibiotroph	16 (6689)	This study
*Mycosphaerella graminicola*	Fungi	Hemibiotroph	48 (10,952)	This study
*Phytophthora infestans*	Chromista	Hemibiotroph	21 (22,658)	This study
*Macrophomina phaseolina*	Fungi	Necrotroph	31 (14,249)	This study
*Fusarium graminearum*	Fungi	Necrotroph	63 (13,321)	Liu et al. [1]
*Fusarium oxysporum*	Fungi	Necrotroph	53 (17,701)	Liu et al. [1]
*Botrytis cinerea*	Fungi	Necrotroph	40 (16,448)	Liu et al. [1]
*Cryptococcus neoformans*	Fungi	Animal pathogen	19 (6431)	Liu et al. [1]
*Histoplasma capsulatum*	Fungi	Animal pathogen	29 (8038)	This study
*Coccidioides immitis*	Fungi	Animal pathogen	38 (10,457)	This study
*Candida albicans*	Fungi	Animal pathogen	21 (6185)	Liu et al. [1]
*Aspergillus fumigatus*	Fungi	Animal pathogen	40 (9929)	This study
*Aspergillus nidulans*	Fungi	Saprotroph	55 (10,658)	Liu et al. [1]
*Neurospora crassa*	Fungi	Saprotroph	35 (9935)	This study
*Podospora anserina*	Fungi	Saprotroph	40 (10,956)	This study
*Saccharomyces cerevisiae*	Fungi	Saprotroph	11 (6713)	This study
*Schizosaccharomyces pombe*	Fungi	Saprotroph	12 (5058)	This study
*Phanerochaete chrysosporium*	Fungi	Saprotroph	60 (13,602)	This study
*Serpula lacrymans*	Fungi	Saprotroph	39 (14,495)	This study
*Laccaria bicolor*	Fungi	Symbiotic	95 (23,130)	This study
*Arabidopsis thaliana*	Viridiplantae	N/A	568 (35,386)	Kuroda et al. [31]
*Oryza sativa japonica*	Viridiplantae	N/A	687 (67,393)	Jain et al. [2]
*Cicer arietinum*	Viridiplantae	N/A	285 (28,269)	Gupta et al. [3]

### Basic structure and localization

Information of protein length was gathered from the NCBI database (*http://**www.ncbi.nlm.nih.gov*). General feature format (GFF) data were used to identify exon–intron structures of all F-box domain-containing proteins from the *M. phaseolina* fungus with the help of online software Gene Structure Display Server 2.0 (*http://gsds.cbi.pku.edu.cn/*). Online-based software WoLF PSORT was applied to predict the probable localization of all thirty-one F-box box proteins [32]. Different domains were identified and analyzed with the online software SMART (*http://smart.embl.de/*) and Pfam (*https://pfam.xfam.org/*).

### Sequence alignment and chromosomal mapping of F-box proteins

Protein sequences were collected from two different databases (*http://**www.ncbi.nlm.nih.gov* and *http://cfgp.riceblast.snu.ac.kr*), and those sequences were aligned using Clustal Omega software (https://www.ebi.ac.uk/Tools/msa/clustalo/). The physical locations of *Macrophomina phaseolina* F-box proteins on respective chromosomes/ scaffolds were identified using BLASTN search against the local *Macrophomina phaseolina* database as the *Macrophomina phaseolina* was not assembled at chromosome-scale so assembled sequences in the form of scaffolds were used for chromosomal mapping. The starting position of each protein was shown on the chromosome or scaffolds.

### Gene ontology (GO) analysis

Gene ontology (GO) annotation of F-box proteins for understanding the biological processes, cellular components and molecular functions were done through Blast2GO program (*https://www.biobam.com/download-omicsbox/*).

### Phylogenetic analysis

The full-length amino acid sequences of putative F-box proteins of stem rot fungus *M. phaseolina*, and published F-box proteins from different fungal organisms were collected from the CFGP 2.0 (*http://cfgp.riceblast.snu.ac.kr*) [30]. In order to understand the relationship among the F-box proteins in *M. phaseolina* and the selective fungal organism, a phylogenetic tree was constructed by MEGA6 software. Initially, multiple-sequence alignment of abovementioned fungal species of F-box protein sequences were created using the ClustalW tool in MEGA6, and then according to the alignment file, a phylogenetic tree was generated using the neighbor-joining (NJ) method [33] inferred from 1000 bootstrap replicates with other default parameter.

## Results

### Identification of F-box proteins in different fungi

**Fig. 1:**
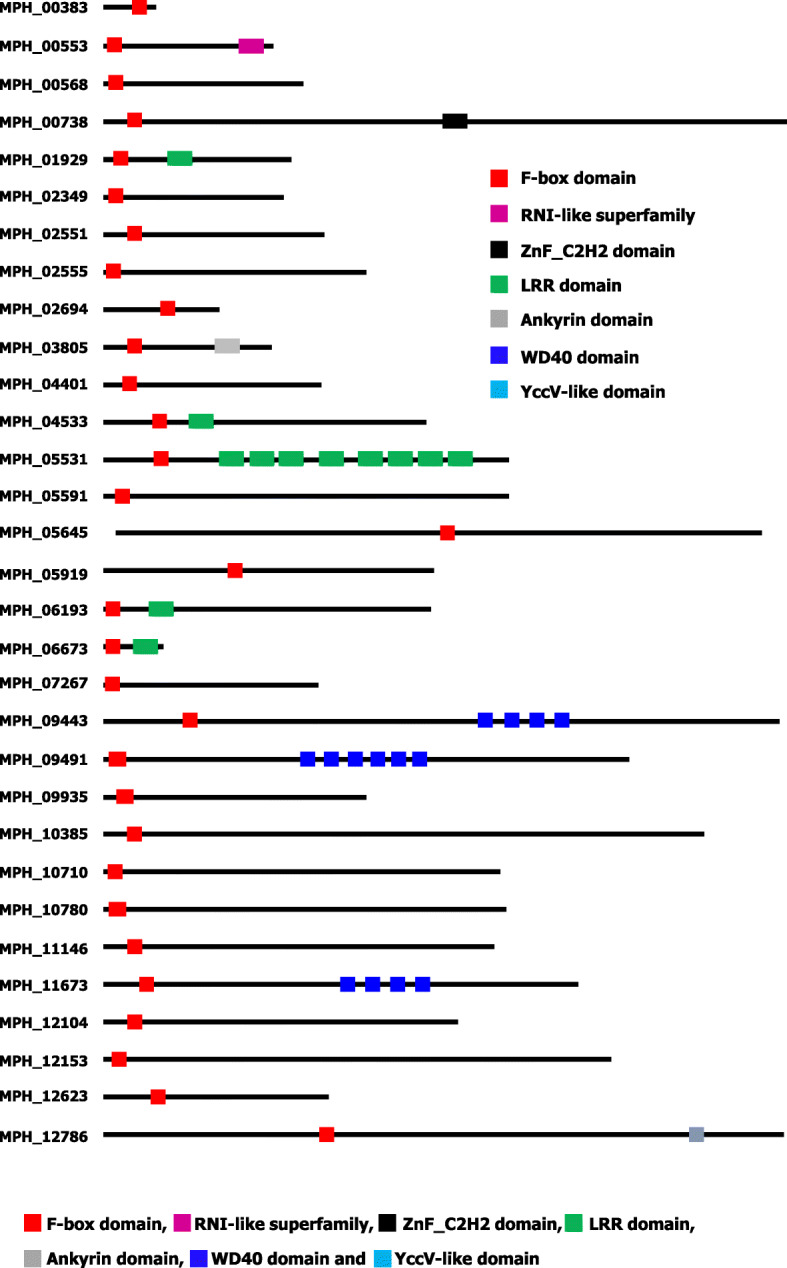
Schematic diagram of F-box protein with different motifs in the stem rot fungus *M. phaseolina*

Members of the F-box protein family consists of a large number of proteins having the F-box domain as a signature. To identify the F-box proteins from different economically important fungi including stem rot fungus *Macrophomina phaseolina* several InterPro domains were used. In this analysis, the F-box protein from model plants *Arabidopsis thaliana, Cicer arietinum*, *Oryzae sativa*, and model fungus rice blast fungus (*Magnaporthe oryzae*) were also added for the comparison analysis with the F-box proteins from the identified fungal genomes [2, 13, 22, 31]. The genome-wide search found great variations of fungal F-box proteins along with plants where thirty one F-box domain-containing protein was found in jute stem rot fungus *M. phaseolina* genome (Table 1 and Table S1). *Saccharomyces cerevisiae, Schizosaccharomyces pombe*, *Ustilago maydis*, and *Puccinia graminis* contain 11, 12, 16, and 17, respectively, which were lower in number, whereas *Fusarium graminearum*, *Phanerochaete chrysosporium*, and *Aspergillus nidulans* contain a higher number of F-box protein (Table 1). Domain analysis detected several other functional domains like WD40, LRR, and ankyrin along with F-box domain in the *M. phaseolina* fungus (Fig. 1). This result might indicate the interacting protein variations in the *M. phaseolina* fungus.

### Basic structure and chromosomal distribution of *M. phaseolina* F-box proteins

**Fig. 2:**
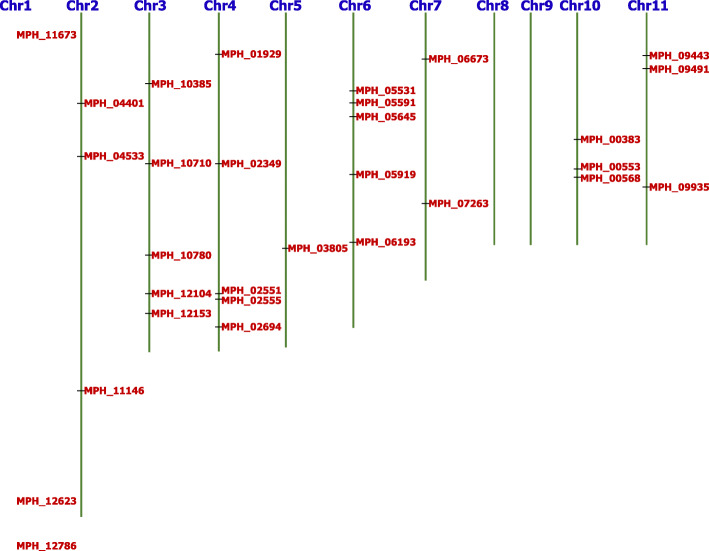
Chromosomal distribution of *Macrophomina phaseolina* F-box protein

Protein structures provide the possible function of that particular protein as well as indicate the origin of that particular gene in a genome. To predict the probable functions of F-box proteins, basic structure was analyzed and summarized in Table 2. Analysis found that > 50% of the identified F-box proteins did not have any intron in their protein structure. In addition, another 23% protein contained two exons and one intron in their protein sequences. It was also found that two proteins namely MPH_10780 and MPH_12786 had eight and twelve exons, respectively; however, these two proteins did not have the highest protein length among the rest of the F-box proteins. MPH_00738 protein showed the highest protein length having only four exons and three introns in the protein structure (Table 2 and Figure S1). Around 55% and 33% F-box proteins were found to be localized in the cytoplasm and nucleus, respectively. However, only two proteins were predicted to localize in the mitochondria and endoplasmic reticulum (ER), respectively. From the localization analysis, it was confirmed that most of the F-box proteins were localized inside the cell, and it will help to design specific antifungal chemicals. Chromosomal locations of the F-box proteins were determined using the draft genome sequences of *Macrophomina phaseolina* [29]. Thirty [30] F-box proteins were located on nine (09) different chromosomes (Fig. 2). Chromosomal position of MPH_00738 was not found, and chromosomes 8 and 9 were not found to contain any F-box protein. From this result, no cluster of F-box protein was observed, and it indicated that gene duplication event might not have occurred for this protein family in *Macrophomina phaseolina*.

**Table 2:** Basic information of F-box protein in the stem rot fungus *Macrophomina phaseolina*

Gene locus	Nucleotide size	Protein size	Exon	Intron	Localization
MPH_00383	360	120	1	0	Cytoplasmic
MPH_00553	1191	397	2	1	Cytoplasmic
MPH_00568	1389	463	1	0	Cytoplasmic
MPH_00738	4842	1614	4	3	Nuclear
MPH_01929	1338	446	1	0	Cytoplasmic
MPH_02349	1278	426	1	0	Cytoplasmic
MPH_02551	1560	520	4	3	Cytoplasmic
MPH_02555	1857	619	3	2	Cytoplasmic
MPH_02694	825	275	1	0	Nuclear
MPH_03805	1194	398	1	0	Cytoplasmic
MPH_04401	1536	512	1	0	Cytoplasmic
MPH_04533	1680	560	5	4	Cytoplasmic
MPH_05531	2112	704	1	0	Nuclear
MPH_05591	984	328	3	2	Cytoplasmic
MPH_05645	3387	1129	1	0	Cytoplasmic
MPH_05919	1707	569	2	1	Nuclear
MPH_06193	1152	384	2	1	Cytoplasmic
MPH_06673	339	113	1	0	Nuclear
MPH_07267	1113	371	1	0	Cytoplasmic
MPH_09443	3528	1176	1	0	Nuclear
MPH_09491	1872	624	1	0	Nuclear
MPH_09935	924	308	1	0	Cytoplasmic
MPH_10385	2154	718	2	1	Mitochondrial
MPH_10710	1407	469	2	1	Nuclear
MPH_10780	1455	485	8	7	Nuclear
MPH_11146	1413	471	1	0	Nuclear
MPH_11673	1695	565	2	1	Mitochondrial
MPH_12104	1263	421	3	2	Extracellular
MPH_12153	1809	603	2	1	Extracellular
MPH_12623	798	266	1	0	Cytoplasmic
MPH_12786	2415	805	12	11	Cytoplasmic

### Sequence alignment of *M. phaseolina* F-box proteins

**Fig. 3:**
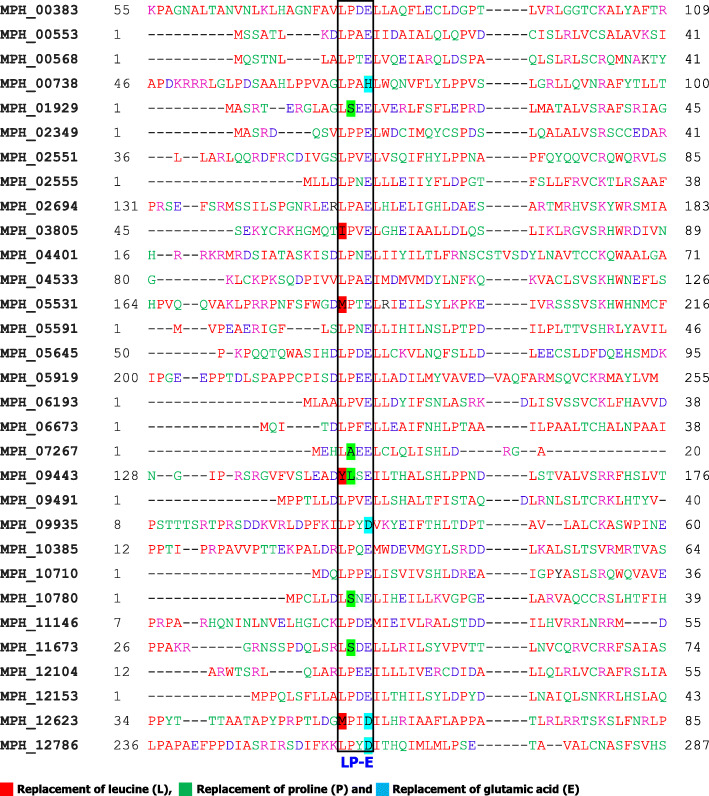
Sequence alignment of F-box protein in the stem rot fungus *M. phaseolina*

To gain the extensive understanding of F-box proteins in the jute stem rot fungus *M. phaseolina* genome, amino acids of all thirty-one proteins were aligned using Clustal Omega (*https://www.ebi.ac.uk/Tools/msa/clustalo/*). Alignment analysis found very low amino acid sequence similarities among the F-box proteins of *M. phaseolina* except the three amino acids leucine (L), proline (P), and glutamic acid (E) (Fig. 3). However, MPH_09443 proteins did not contain either leucine (L) or proline (P), but solely contained glutamic acid (E), and MPH_12623 proteins had only proline (P), and the other two, leucine (L) and glutamic acid (E), were absent in the F-box domain (Fig. 3). It was also observed that leucine (L) replaced with isoleucine (I), methionine (M), tyrosine (Y), and proline (P) changed with alanine (A), serine (S), and leucine (L) in several proteins. However, glutamic acid (E) only altered with the aspartic acid (D) in three F-box proteins. This might be due to the point mutation in the nucleic acid of these proteins in the *M. phaseolina* fungus. BLAST results found very low sequence coverage (36% or less) among the F-box proteins with default e-value (Table S2), pointing that the F-box proteins of *Macrophomina phaseolina* fungus were not duplicated and those are independent proteins.

### Classification of *M. phaseolina* F-box proteins

**Fig. 4:**
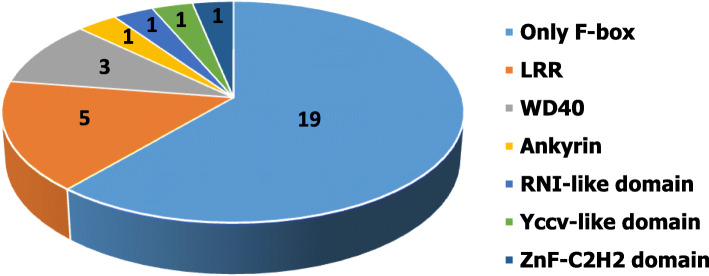
Classification of F-box proteins in *M. phaseolina.* The number of F-box proteins were classified and shown in numbers

Based on the presence of different functional domains along with F-box domain, F-box proteins of *M. phaseolina* fungus were classified. For this analysis, F-box proteins were analyzed using the online software SMART (*http://smart.embl.de/*) and Pfam (*https://pfam.xfam.org/*). Analysis found that five major subfamilies where nineteen proteins contained only F-box domain and did not have any other functional domain in their C-terminal region (Fig. 4). In addition, five proteins and three proteins contained additional leucine-rich repeats (LRR) and WD40 repeats, respectively along with the both F-box domain. Moreover, ankyrin repeat was found as an additional functional domain with the F-box domain in one protein namely MPH_03805. Three proteins (MPH_00553, MPH_00738, and MPH_12153) contained 3 different additional domains like RNI-like, ZnF-C_2_H_2_, and YccV-like along with F-box domain (Table S1). However, no protein was found with an additional domain of unknown function as commonly found in F-box protein of plants genome. This classification might indicate the protein–protein interaction of the additional domain for disease development in *M. phaseolina*. A gene ontology analysis was also carried out to predict the possible functions of *Macrophomina phaseolina* F-box proteins. Analysis found that most of the F-box proteins (28 proteins) involved protein binding rather than molecular function, biological process, and cellular component (Table S3).

### Phylogenetic analysis of F-box proteins

**Fig. 5:**
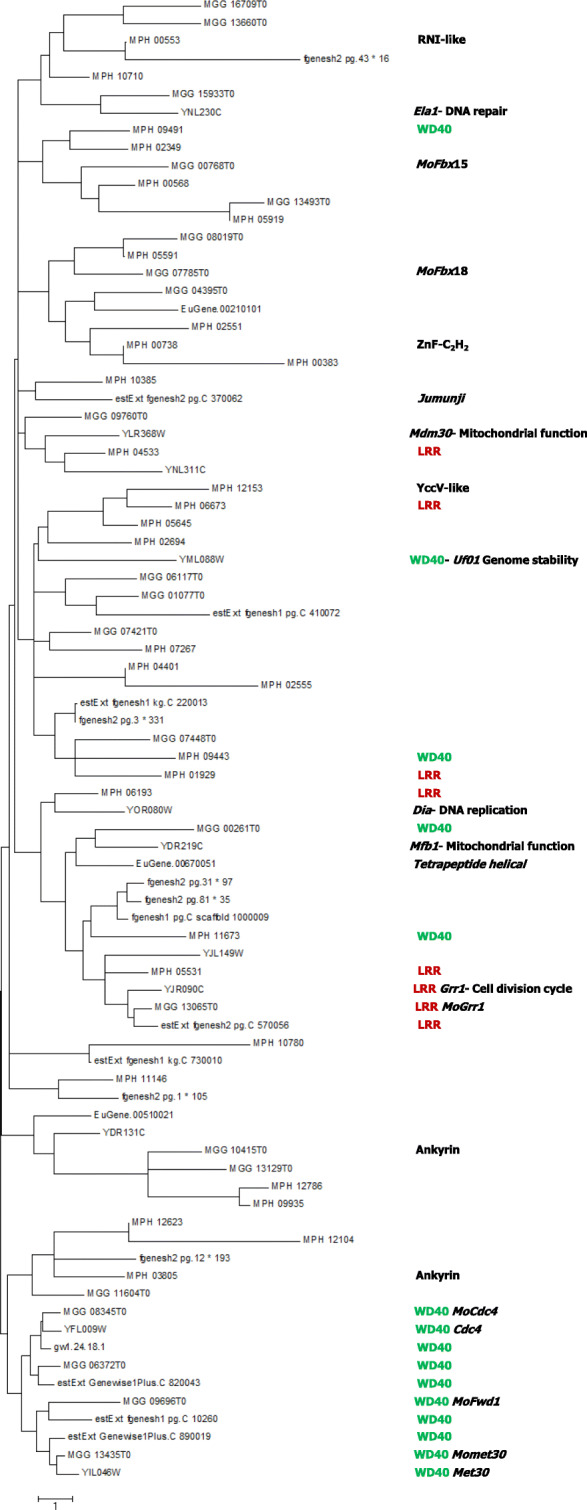
Phylogenetic analysis of F-box proteins of *Macrophomina phaseolina.* Phylogenetic analysis was carried out by MEGA 6.0 program

By using protein sequences, phylogenetic tree was constructed to understand the relationship of F-box proteins in different phytopathogenic fungus. The phylogenetic tree was made through the neighbor-joining approach through MEGA 6.0. It has been found that most of the F-box proteins were highly diverse from one protein to another (Fig. 5). *M. phaseolina* genome contains five F-box protein namely MPH_04533, MPH_06673, MPH_01929, MPH_06193, and MPH_05531 having leucine-rich repeat (LRR); however, MPH_05531 only showed a close relationship with yeast *Grr1*, rice blast fungus *MoGrr1*, and powdery mildew pathogen *B. graminis* (estExt fgenesh2 pg. C 570056), WD40 repeat containing F-box proteins were mostly found in a similar place in the phylogenic tree; however, WD40 repeat containing F-box proteins of *M. phaseolina* seemed to diverse from them and found far away from the major subclade of WD40 domain-containing F-box proteins (Fig. 5). Among the four fungal species, *M. phaseolina* and *M. oryzae* had one ankyrin repeat-containing F-box protein in each fungus, but they are phylogenetically distant and present in different places in the tree indicating the possibility of interaction with different proteins. From the phylogenetic tree, it can also be predicted that sequence diversity of those proteins may lead to diverge functions in fungi.

### Comparison of *M. phaseolina* F-box proteins with other phytopathogenic fungal F-box proteins

It has been reported that protein families vary from organism to organism, and this might help to predict their involvement of physiological process as well as evolution of that particular protein families. In this analysis, F-box proteins of fungi having different lifestyles, and plants were also included to understand the distribution of F-box proteins in different organisms.

**Fig. 6:**
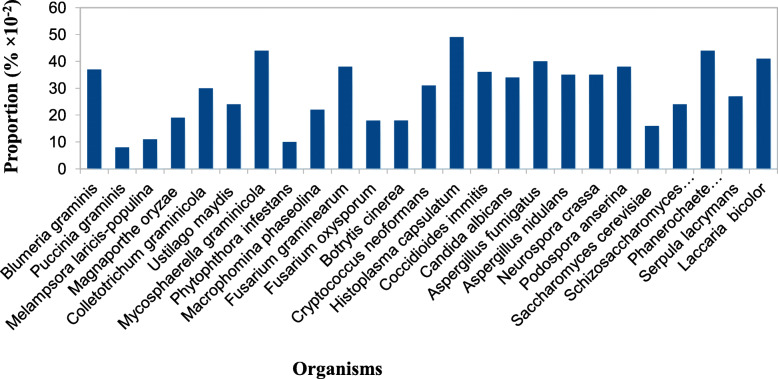
Comparative analysis of F-box proteins in different economically important fungi

Proportion of contained F-box proteins in most selected fungal genomes ranging from 10 to 49% in *Phytophthora infestans* and *Histoplasma capsulatum*, respectively (Fig. 6). It is quite interesting that a proportion of F-box proteins in the kingdom Viridiplantae were much higher than the selected fungi used in this study (Table 1). This result might suggest that plants require more F-box proteins for performing various physiological processes to complete their full life cycle, whereas, a lower number of proteins in fungi indicate their importance during the disease development process in the host. Analysis also revealed that presence of F-box proteins had no relationship with the lifestyles (biotrophic, hemibiotrophic, and necrotrophic nature) and also with the host range of selected fungi used in this study. Symbiotic fungus *Laccaria bicolor* genome contained the highest number of F-box proteins compared with the rest of the fungi; however, the proportions were almost similar with the proportion of other three fungi namely, *Mycosphaerella graminicola, Phanerochaete chrysosporium*, and *Aspergillus fumigatus* (Table 1 and Fig. 6).

**Fig. 7:**
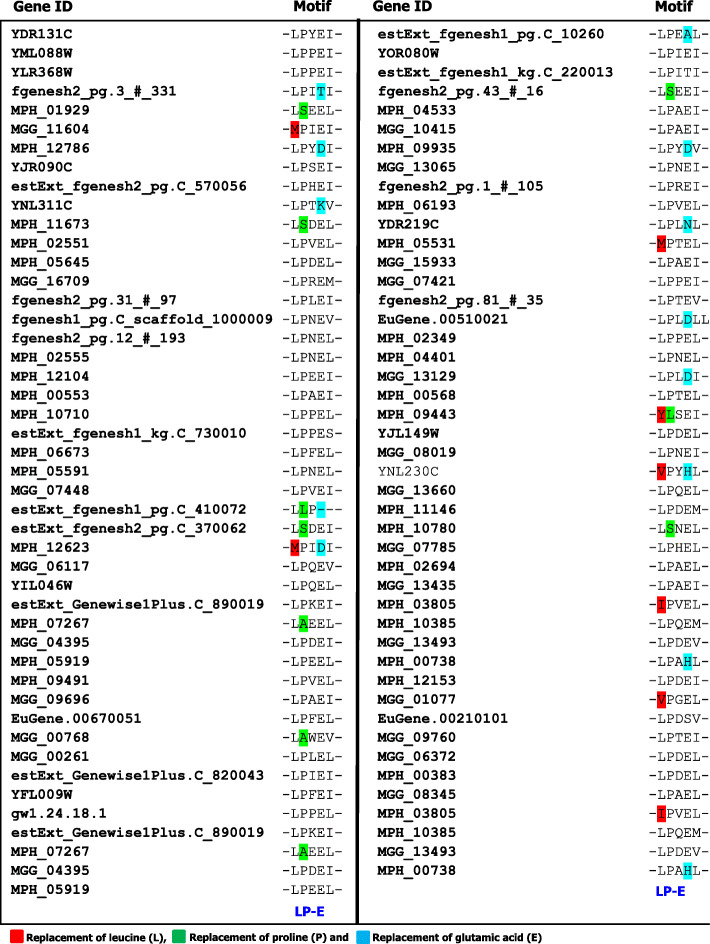
Sequence alignment of F-box protein in four different fungi *(M. phaseolina*, *M. oryzae*, *B. graminis*, and *S. cerevisiae)*

Comparative analysis of sequence alignment found the similar replacement of leucine (L), proline (P), and glutamic (E) are were also observed in the F-box protein of *M. oryzae*, *S. cerevisiae,* and *B. graminis* (Fig. 7). In yeast, *S. cerevisiae*, leucine (L) and proline (P) were not replaced; however, glutamic acid (E) was replaced by lysine (K), asparagine (N), and leucine (L). In *M. oryzae,* leucine (L) was replaced by the valine (V), methionine (M), and proline (P) was replaced by alanine (A). In *B. graminis*, proline (P) was only replaced by the serine (S). However, glutamic acid (E) was replaced by the aspartic acid (D), alanine (A), threonine (T), and serine (S). These results clearly indicate that point mutation is a common event in the living organisms.

SCF complex of F-box protein (skp2), was highly conserved in most selected biotrophic, hemibiotroph, necrotrophic, and symbiotic fungi (data not shown). This result suggests that phosphorylation process is common in all fungi for their growth and development including disease development. Interestingly, fungi contained comparatively more WD40 repeat than the plant F-box protein suggesting that fungi might require more WD40 for disease development (Table 3). Leucine-rich repeats (LRR) were highly present in plants than the fungi, indicating the importance of LRR for physical growth for survival. Abundance of ankyrin repeat in necrotrophic fungi gives hint that this repeat might be involved in the protein–protein interaction for disease development; however, this function has not been yet reported.

**Table 3:** Comparison of leucine-rich repeat (LRR), WD40 (WD), and ankyrin (Ank) repeat in fungi

Species	Kingdom	Phylum	Lifestyle	LRR	WD	Ank
*Blumeria graminis*	Fungi	Ascomycota	Biotroph	3	6	0
*Puccinia graminis*	Fungi	Basidiomycota	Biotroph	0	6	0
*Melampsora laricis-populina*	Fungi	Basidiomycota	Biotroph	1	5	0
*Magnaporthe oryzae*	Fungi	Ascomycota	Hemibiotroph	1	6	1
*Colletotrichum graminicola*	Fungi	Ascomycota	Hemibiotroph	2	6	0
*Ustilago maydis*	Fungi	Basidiomycota	Hemibiotroph	1	7	0
*Mycosphaerella graminicola*	Fungi	Ascomycota	Hemibiotroph	3	9	0
*Phytophthora infestans*	Chromista	Oomycota	Hemibiotroph	0	0	1
*Macrophomina phaseolina*	Fungi	Ascomycota	Necrotroph	5	3	1
*Fusarium graminearum*	Fungi	Ascomycota	Necrotroph	3	8	1
*Fusarium oxysporum*	Fungi	Ascomycota	Necrotroph	3	7	2
*Botrytis cinerea*	Fungi	Ascomycota	Necrotroph	1	6	0
*Cryptococcus neoformans*	Fungi	Basidiomycota	Animal pathogen	1	10	0
*Histoplasma capsulatum*	Fungi	Ascomycota	Animal pathogen	3	9	0
*Coccidioides immitis*	Fungi	Ascomycota	Animal pathogen	2	7	4
*Candida albicans*	Fungi	Ascomycota	Animal pathogen	0	3	0
*Aspergillus fumigatus*	Fungi	Ascomycota	Animal pathogen	2	8	2
*Aspergillus nidulans*	Fungi	Ascomycota	Saprotroph	2	10	2
*Neurospora crassa*	Fungi	Ascomycota	Saprotroph	2	7	2
*Podospora anserina*	Fungi	Ascomycota	Saprotroph	2	8	3
*Saccharomyces cerevisiae*	Fungi	Ascomycota	Saprotroph	0	3	0
*Schizosaccharomyces pombe*	Fungi	Ascomycota	Saprotroph	1	7	0
*Phanerochaete chrysosporium*	Fungi	Basidiomycota	Saprotroph	1	6	0
*Serpula lacrymans*	Fungi	Basidiomycota	Saprotroph	0	7	0
*Laccaria bicolor*	Fungi	Basidiomycota	Symbiotic	0	11	0
*Arabidopsis thaliana*	Viridiplantae	Streptophyta	N/A	29	2	ND
*Oryza sativa japonica*	Viridiplantae	Streptophyta	N/A	61	2	ND
*Cicer arietinum*	Viridiplantae	Streptophyta	N/A	39	4	ND

Note: *LRR* leucine-rich repeat, *WD* WD40 domain, *Ank* ankyrin repeat, *ND* not determined

## Discussion

Proteolysis is not only a common process for living organisms but also necessary for protein homeostasis for proper growth and development through the cell division cycle [34]. Several components have been reported to be involved in the proteolysis, and a novel motif called F-box is responsible for the ubiquitin-mediated proteolysis [14].

F-box proteins are highly species-specific, and there might be no relationship between the organism’s genome size with their proportion. Protein number can be changed with the protein loss and gain in the genome [19, 35]. In this research, fungal genome was found to contain different numbers of F-box proteins (Table 1); however, there was no distinct pattern for existing of F-box proteins. The number of F-box proteins in fungal genome was comparatively much lower than the plant genome except symbiotic fungus *Laccaria bicolor*. Interestingly, the proportion of fungal F-box protein was much smaller than the proportion of plant F-box protein (data not shown). It seemed that fungi need less number of F-box protein for their survival and disease development. This prediction is supported by the research where 24 F-box proteins were identified in rice blast fungus (*M. oryzae*) genome; however, only three F-box proteins were found to be involved for full virulence [22]. Our identified F-box protein number might be varied because of the parameters (default) that we selected for our BLAST search against the F-box domain. It has been reported that BLAST is sensitive enough to identify the sequences from the remote homologous protein [36].

Exon–intron configuration of the F-box proteins has a distinct feature of having intron-less protein in many plant genomes [37]. Domain arrangement and composition can be resulted through shifting of exon–intron as well as insertion and/ or deletion of exon [19]. Stem rot fungal (*M. phaseolina*) F-box proteins contained > 50% of the total intron-less protein (Table 2). Although it is not clear how these proteins arisen in the stem rot fungus *M. phaseolina* genome, it seems to be originated through gene duplication or reverse transcription and integration. It has been reported that the number of genes in eukaryotic organisms can be duplicated through natural selection as well as by reverse transcription and then integration [38, 39]. It was also reported that intron/exon structure of a subfamily had a strong structural relationship between the chickpea F-box proteins [13].

Leucine (L) and proline (P) were reported as the compulsory amino acids for the function of F-box protein in the living organisms [15]. However, leucine (L), proline (P), and glutamic acid (E) were found to be replaced by several other amino acids in different F-box proteins in other four fungi (Fig. 3 and 7). These changes of amino acid might occur due to the single nucleotide substitution during the replication of DNA. It has been reported that single nucleotide replacement for another can occur during the replication of DNA [40, 41]. Amino acid replacement can also create mutational fore in living organisms [42]. It was hypothesized that alteration of amino acid in F-box proteins might lead to diverse functions in fungi. It also reports that due to the amino acid substitution, the similar gene showed diverse function in insulin delivery and reduction of enzymatic activity in human [43, 44].

Protein domains are the small units of evolution as well as the basic components of protein structure and function [45, 46]. In general, fungi contain various numbers of domain compared with the other organisms [47]. Expansion of domain in the fungal genome occurred by domain duplication through recombination [48]. F-box proteins contain generally one or more variable protein–protein interacting domain such as leucine-rich repeat (LRR), kelch repeat, WD40 repeat, and more for interaction with the target protein [49]. Domain analysis revealed that stem rot fungus contains a large fraction (61%) of F-box proteins having only F-box domain (Fig. 4). It is highly likely that many of the F-box proteins might evolve into new ones in the stem rot fungus which had not undergone domain expansion yet or lost the domain for functional losses. In addition, conservation of F-box domain, skp2-like in fungi indicated the dependency of phosphorylation; however, proper–protein degradation relies on the multiple mechanisms [50].

Phylogenetic analysis not only shows the relation among the proteins but also indicate their evolutionary histories including climatic and geographical history on earth [51]. Each fungal F-box proteins were highly divergent in their sequences from others even though they had the similar functional domain (Fig. 5). It was expected that phylogenetically closely located proteins with the similar domain are involved in a similar function to interact with a similar substrate. However, same domain proteins might involve in different functions due to its point mutation in proteins sequences [52]. Similar event in domain shuffling for protein diversification was reported in rice where individual duplication was found [53]. In this paper, genome-wide F-box proteins have been identified in the jute stem rot fungus *M. phaseolina* that have been believed to be involved in protein degradation.

## Conclusion

In this experiment, thirty-one F-box proteins from the jute stem rot fungus *M. phaseolina* were identified and analyzed. Based on the existence of different domains, all proteins were categorized in five groups. Large group (61%) consists of F-box domain alone; however, leucine-rich repeat (LRR) and WD40-containing group were in the second and third largest group, respectively. Single nucleotide substitution resulted in leucine (L), proline (P), and glutamic acid (E) in several F-box proteins in different fungi. Phylogenetic tree revealed that proteins from the same group are highly diverse from each other indicating the diverse functions of F-box proteins in fungi. These results provide an essential understanding of F-box protein in *M. phaseolina* and constitute a strong foundation for further investigation in the regulation of fungal virulence, which may lead to novel approaches in developing new antifungal agents.

## Supplementary Information

**Additional file 1: Table S1.** Identified F-box proteins in the stem rot fungus *Macrophomina phaseolina*.

**Additional file 2: Table S2.** BLAST search result of F-box protein of *Macrophomina phaseolina*.

**Additional file 3: Table S3.** BLAST2GO analysis of Macrophomina phaseolina F-box proteins.

**Additional file 4: Figure S1.** Exon-intron structure and length F-box protein in stem rot fungus *M. phaseolina*.

## Data Availability

All the protein sequences are available in the NCBI database.

## Abbreviations

DNA: Deoxyribonucleic acid
ATP: Adenosine triphosphate
SCF: Skp, Cullin and F-box containing
Grr1: Glucose repression resistant 1
BARJ: Basic and Applied Research on Jute Project
NCBI: National Center for Biotechnology Information
CFGP: Comparative fungal genomic platform
GFF: General feature format
MEGA: Molecular Evolutionary Genetics Analysis
NJ: Neighbor-joining
LRR: Leucine-rich repeat
ER: Endoplasmic reticulum
