# Supplementary material for: Genome-wide identification of F-box proteins in Macrophomina phaseolina and comparison with other fungus
Source: J Genet Eng Biotechnol. 2021 Mar 24;19:46. doi: 10.1186/s43141-021-00143-0 (PMC7991009; doi:10.1186/s43141-021-00143-0)
Supplement: Supplementary file 1 — Additional file 1: Table S1. Identified F-box proteins in the stem rot fungus Macrophomina phaseolina. [file 43141_2021_143_MOESM1_ESM.docx]

Table S1. Identified F-box proteins in the stem rot fungus *Macrophomina phaseolina*

| **Protein** | **Name of the protein** | **Additional domain** |
| --- | --- | --- |
| MPH_00383 | F-box |  |
| MPH_00553 | F-box | RNI-like superfamily |
| MPH_00568 | F-box |  |
| MPH_00738 | F-box | ZnF-C_2_H_2_ |
| MPH_01929 | F-box | LRR |
| MPH_02349 | F-box |  |
| MPH_02551 | F-box |  |
| MPH_02555 | F-box |  |
| MPH_02694 | F-box |  |
| MPH_03805 | F-box | Ankyrin |
| MPH_04401 | F-box |  |
| MPH_04533 | F-box | LRR |
| MPH_05531 | F-box | LRR |
| MPH_05591 | F-box |  |
| MPH_05645 | F-box |  |
| MPH_05919 | F-box |  |
| MPH_06193 | F-box | LRR |
| MPH_06673 | F-box | LRR |
| MPH_07267 | F-box |  |
| MPH_09443 | F-box | WD40 |
| MPH_09491 | F-box | WD40 |
| MPH_09935 | F-box |  |
| MPH_10385 | F-box |  |
| MPH_10710 | F-box |  |
| MPH_10780 | F-box |  |
| MPH_11146 | F-box |  |
| MPH_11673 | F-box | WD40 |
| MPH_12104 | F-box |  |
| MPH_12153 | F-box | YccV-like |
| MPH_12623 | F-box |  |
| MPH_12786 | F-box |  |
