# Supplementary material for: Genome-wide identification of F-box proteins in Macrophomina phaseolina and comparison with other fungus
Source: J Genet Eng Biotechnol. 2021 Mar 24;19:46. doi: 10.1186/s43141-021-00143-0 (PMC7991009; doi:10.1186/s43141-021-00143-0)
Supplement: Supplementary file 4 — Additional file 4: Figure S1. Exon-intron structure and length F-box protein in stem rot fungus M. phaseolina. [file 43141_2021_143_MOESM4_ESM.pptx]

## Slide 1
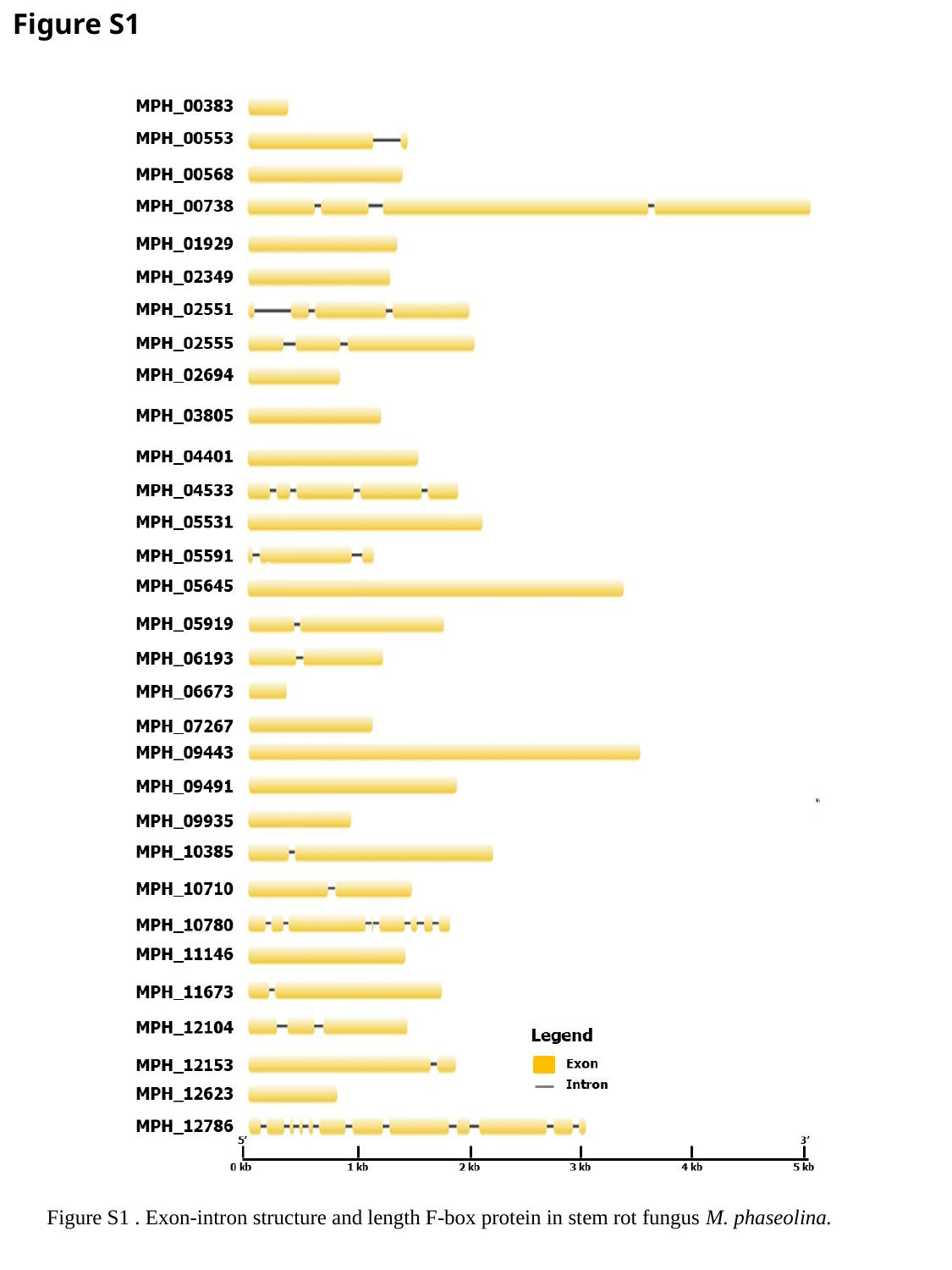

Figure S1
Figure S1 . Exon-intron structure and length F-box protein in stem rot fungus M. phaseolina.
